# Printing tissue-engineered scaffolds made of polycaprolactone and nano-hydroxyapatite with mechanical properties appropriate for trabecular bone substitutes

**DOI:** 10.1186/s12938-023-01135-6

**Published:** 2023-07-20

**Authors:** Zahra Yazdanpanah, Nitin Kumar Sharma, Alice Raquin, David M. L. Cooper, Xiongbiao Chen, James D. Johnston

**Affiliations:** 1 Division of Biomedical Engineering, College of Engineering, University of Saskatchewan, Saskatoon, SK Canada; 2 Department of Mechanical Engineering, College of Engineering, University of Saskatchewan, Saskatoon, SK Canada; 3 Institut Catholique Des Arts Et Métiers, 85000 La Roche-Sur-Yon, France; 4 Department of Anatomy, Physiology, and Pharmacology, College of Medicine, University of Saskatchewan, Saskatoon, SK Canada

**Keywords:** Polycaprolactone, Nano-hydroxyapatite, Three-dimensional printing, Bone scaffold, Lattice structure, Staggered structure

## Abstract

**Background:**

Bone tissue engineering, based on three-dimensional (3D) printing technology, has emerged as a promising approach to treat bone defects using scaffolds. The objective of this study was to investigate the influence of porosity and internal structure on the mechanical properties of scaffolds.

**Methods:**

We fabricated composite scaffolds (which aimed to replicate trabecular bone) from polycaprolactone (PCL) reinforced with 30% (wt.) nano-hydroxyapatite (nHAp) by extrusion printing. Scaffolds with various porosities were designed and fabricated with and without an interlayer offset, termed as staggered and lattice structure, respectively. Mechanical compressive testing was performed to determine scaffold elastic modulus and yield strength. Linear regression was used to evaluate mechanical properties as a function of scaffold porosity.

**Results:**

Different relationships between mechanical properties and porosities were noted for the staggered and lattice structures. For elastic moduli, the two relationships intersected (porosity = 55%) such that the lattice structure exhibited higher moduli with porosity values greater than the intersection point; vice versa for the staggered structure. The lattice structure exhibited higher yield strength at all porosities. Mechanical testing results also indicated elastic moduli and yield strength properties comparable to trabecular bone (elastic moduli: 14–165 MPa; yield strength: 0.9–10 MPa).

**Conclusions:**

Taken together, this study demonstrates that scaffolds printed from PCL/30% (wt.) nHAp with lattice and staggered structure offer promise for treating trabecular bone defects. This study identified the effect of porosity and internal structure on scaffold mechanical properties and provided suggestions for developing scaffolds with mechanical properties for substituting trabecular bone.

**Supplementary Information:**

The online version contains supplementary material available at 10.1186/s12938-023-01135-6.

## Background

Bone is a resilient tissue with self-healing capacity. However, management of critical-sized defects (CSDs), which result from disease or a trauma, remains a substantial orthopedic challenge given that they cannot be spontaneously healed by the patient’s body and their repair needs surgical intervention [1–4]. Approximately two million bone grafts are annually implanted worldwide to repair bone defects [4, 5]. For instance, repairing a CSD in anatomical sites such as pelvis is important given it mainly consists of low-density trabecular bone covered by a thin layer of high-density cortical bone. Related, traumatic incidents in pelvic bone can be fatal [6]. Current treatments are mainly based on the use of traditional bone grafts such as autografts, allografts, and xenografts [7–9]. However, the clinical usage of traditional treatments has been restricted due to associated drawbacks such as limited donor supply and donor sites, additional surgery, the potential risk of disease transmission, and immune response after implantation [9–13].

Bone tissue engineering (BTE), based on three-dimensional (3D) printing technology, has received increasing attention as a potential remedy to repair bone defects unable to be repaired on their own [4, 14, 15]. For BTE, biomedical scaffolds are a key component to provide a temporary environment for extracellular matrix formation, cellular activity, as well as mechanical support [15, 16]. For scaffold fabrication, appropriate material selection, architectural design, controlled chemistry, and interconnected porosity are key factors in achieving mechanical integrity, proper cellular activity, nutrient delivery/waste removal, bone ingrowth, and vascularization for the specific site of application [17]. Polycaprolactone (PCL) is a widely used synthetic polymer in fabricating scaffolds because of its biocompatibility, high printability, and fast solidification after being extruded [18–22]. However, PCL has low adhesion due to its hydrophobic nature, which results in poor osteo-conduction properties and slow degradation [14, 20]. Bioceramic hydroxyapatite (HAp) has been extensively used as an additive to enhance hydrophilicity, osteoconductivity, and degradation rate of PCL [5, 14, 23]. In addition to biological influence, the load-bearing capacity of PCL can also be improved by adding HAp to the PCL matrix [24, 25]. In light of this, composite scaffolds made of PCL and HAp have been explored in BTE studies to achieve improved mechanical properties and biological functionality [20, 25–28].

Mechanical properties of scaffolds have been found to be highly dependent on the porosity level [29, 30]. A high degree of porosity diminishes the load-carrying capacity of a scaffold but improves fluid penetration (i.e., permeability), facilitating nutrient diffusion, oxygen exchange, and waste removal, thus affecting new bone formation or regeneration [31, 32]. Internal scaffold structure and strand arrangement also affect and regulate the mechanical response and cellular functionality [26, 31]. The use of an interlayer offset, which leads to misaligned/shifted strands within a scaffold, provides better support for cellular activities as the misaligned strands provide higher anchorage points for cells to attach [33, 34]. Conversely, strands in a scaffold without an interlayer offset are directly aligned; thus, there is fewer anchorage points and cells travel in a direct path, which in turn negatively affects cellular activities [33, 35]. From a mechanical point of view, scaffolds containing an interlayer offset exhibit different mechanical properties when compared to scaffolds with no interlayer offset [5, 34, 36], though other studies found no difference [5, 37]. Conflicting findings may be due to different relationships between mechanical properties and strand arrangement (characterized in terms of porosity). To clarify, the strands of a scaffold without an interlayer offset primarily experience compressive loading due to shared supporting points; whereas, strands with an offset primarily experience bending as the supporting points are suspended and are no longer shared [38]. The longer the length between strands (where porosity is high), the greater the effect of bending. As such, different mechanical properties may present with scaffolds having large distances between strands (high porosity), whereas no differences may present with shorter distances (low porosity).

To address conflicting findings in the literature, as well as to advance knowledge regarding scaffold design and mechanics, there is a need to characterize mechanical properties of scaffolds in relation to porosity with and without an interlayer offset. To this end, in this study, the commonly studied lattice structure (without interlayer offsetting) was chosen as the main internal structure as well as staggered structure (with interlayer offsetting) as an emerging approach [32, 39]. The main objective of this study was to derive and compare relationships linking porosity with mechanical properties for lattice and staggered scaffolds. The secondary objective of this study pertained to offering suggestions for the development of PCL/30% (wt.) nHAp scaffolds with desired mechanical properties for substituting native bone. For this study, the target tissue was trabecular bone featured by a minimum compressive elastic modulus of ~ 100 MPa [40] and a minimum compressive yield strength of ~ 2 MPa [41].

## Results

**Fig. 1:**
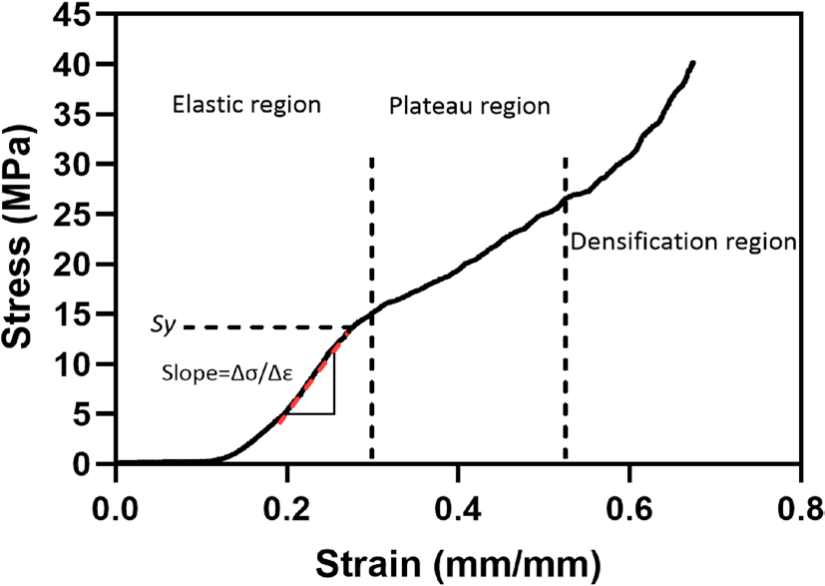
A representative stress–strain curve of PCL/30% (wt.) nHAp after compression test displaying three main regions of elastic, plateau, and densification. The point *Sy* pertains to yield strength and $\frac{\Delta \sigma }{\Delta \varepsilon }$ pertains to the slope of linear stage in the elastic region of stress–strain curve used to derive apparent elastic modulus (*E*)

Representative stress–strain curves obtained from compression tests showed that lattice and staggered scaffolds responded in an elastic–plastic manner and displayed a similar compressive response including three main stages (Fig. 1), regardless of the porosity degree and internal structure tested. The first region (at small strains), which is called a linear elastic region, is attributed to the capability of scaffold’s strands and their junctions in adjacent layers to withstand applied load (i.e., this stage is controlled by strand deformation); 2) the second stage (at medium strains), known as collapse plateau region, is where strand buckling and pore collapse begin; 3) in the third stage (at high strains), which is called densification region, a complete pore collapse throughout the scaffold occurs and strands begin to touch, resulting in a steep increase in stress with increasing compressive strain. In the densification region, scaffolds are no longer porous and they act as a solid structure, which in turn provides high resistance to the applied load [30, 42, 43].

**Fig. 2:**
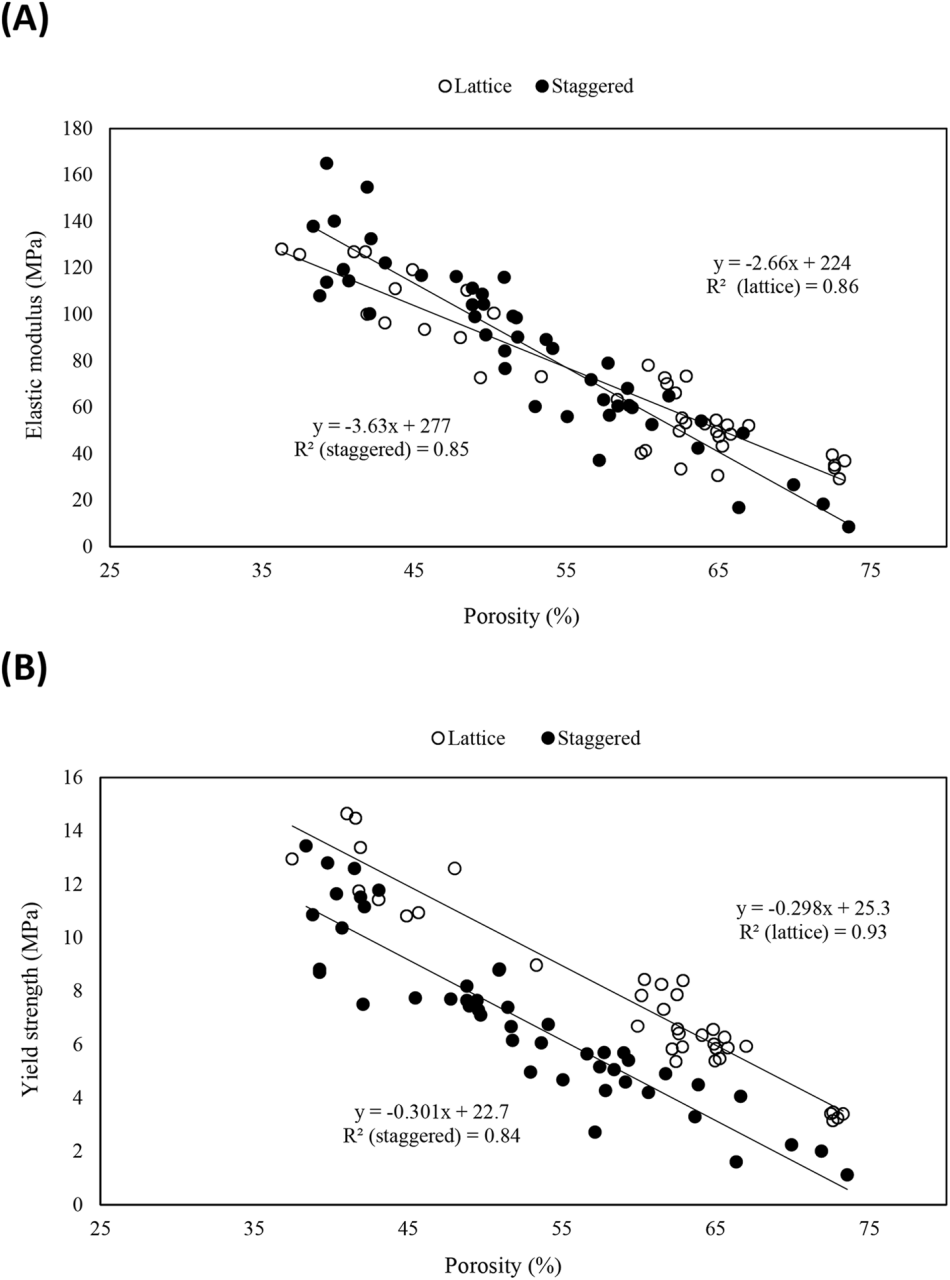
Mechanical properties of scaffolds as a function of porosity: **A** *E* vs. porosity, **B** *Sy* vs. porosity

Regression analysis indicated that porosity explained a high degree of variation in elastic moduli (lattice: *R*^2^ = 0.86; staggered: *R*^2^ = 0.85) and yield strength (lattice: *R*^2^ = 0.93; staggered: *R*^2^ = 0.84). Overall tests of coincidence indicated that the staggered and lattice structures exhibited different modulus–porosity and strength–porosity relationships (*p* ≤ 0.05). For elastic moduli, the two relationships intersected (porosity ≈ 55%) such that the lattice structure exhibited higher moduli with porosity values greater than the intersection point; vice versa for the staggered structure (Fig. 2A). For yield strength, the lattice structure exhibited higher strength for all porosities (Fig. 2B).

Due to 3D printer malfunction, a portion of the staggered scaffolds were 3D printed with 5 layers instead of the intended 6 layers. For verification purposes, supplementary statistical analyses indicated that the 5- and 6-layer staggered scaffolds offered similar mechanical properties and followed the same modulus–porosity and strength–porosity curves (Additional file 1: Figure S1). Accordingly, mechanical testing results from 5- and 6-layer scaffolds were pooled.

**Fig. 3:**
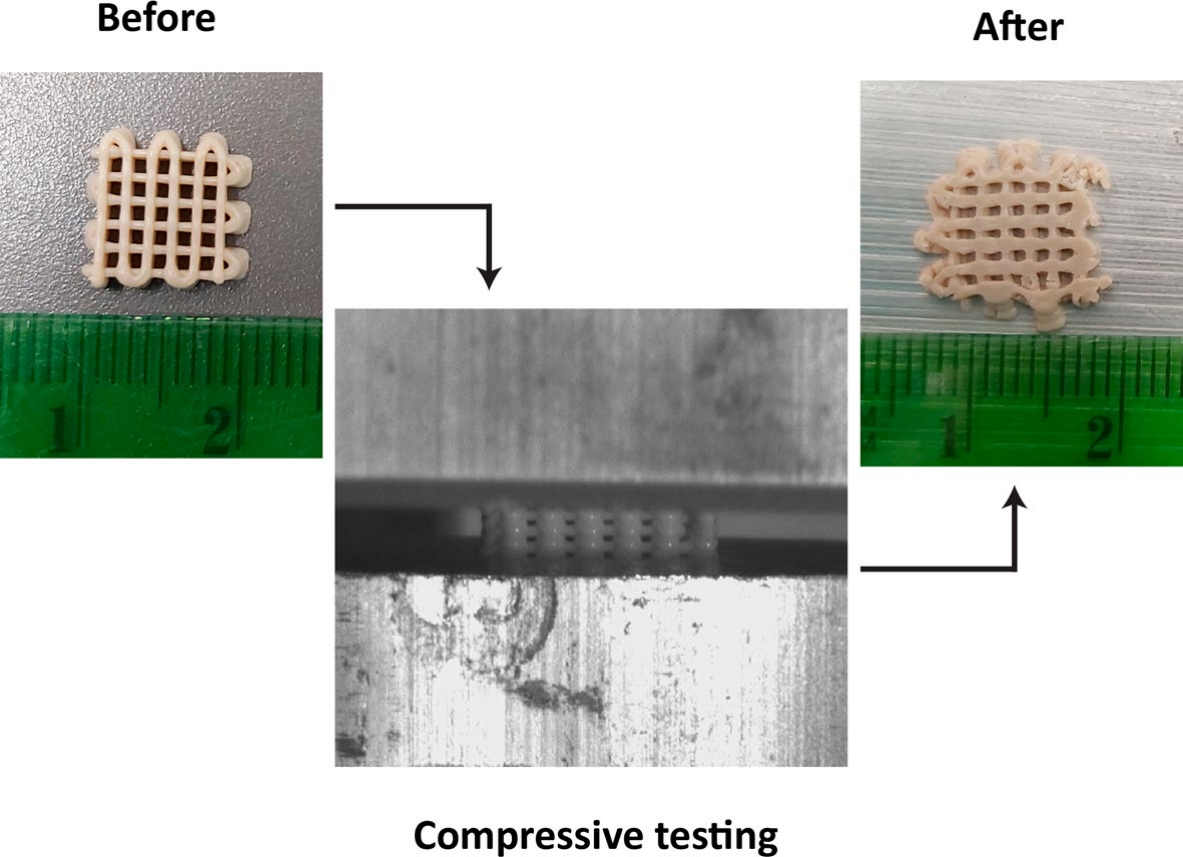
Representative images of a scaffold before and after compressive mechanical testing

Representative images pertaining to a scaffold before and after mechanical testing are displayed in Fig. 3.

## Discussion

This study assessed relationships linking mechanical properties of PCL/30% (wt.) nHAp scaffolds with porosity for different scaffold structures (lattice and staggered). Our results (Fig. 2A, B) indicated that porosity explained a high degree of variation in elastic moduli and yield strength, with the staggered and lattice structures exhibiting different modulus–porosity and strength–porosity relationships. In terms of novel findings, this study appears to explain, for the first time, conflicting findings in the literature related to the mechanical properties of lattice and staggered scaffolds (i.e., some studies reported that lattice scaffolds exhibit superior mechanical properties while other studies found no differences between lattice and staggered scaffolds in terms of elastic modulus). Here different relationships between porosity and mechanical properties appear to explain conflicting findings. Another novel aspect pertains to specific recommendations for designing and constructing lattice and staggered scaffolds with mechanical properties mimicking trabecular bone.

Mechanical findings in this research were in line with previous observations [29, 30, 44], and observed relationships with porosity appear to explain conflicting findings in the literature. To clarify, Park et al. [26] found a lower compressive elastic modulus (−45.5%) in staggered scaffolds of PCL/40% (wt.) HAp with a porosity of ~ 92% when compared to that of lattice scaffolds. Additionally, staggered scaffolds of PCL with a porosity of ~ 95% demonstrated lower elastic modulus (−44%) and yield strength (−48%) than that of lattice scaffolds at the similar porosity [45]. These findings pertained to a high porosity level and mimicked our findings at high porosities. Similarly, staggered scaffolds of polylactic acid (PLA)/polyethylene glycol (PEG) showed lower elastic moduli (−69%) at high porosities (~ 75%) while scaffolds made of PLA/PEG/glass particles exhibited lower elastic moduli (−56%) at high porosities (70%) than lattice scaffolds at similar porosity levels (75% and 70%, respectively) [39]. The findings of Lee et al. [37] though noted no differences in elastic moduli for lattice and staggered structures made from PCL/Poly lactic-co-glycolic acid. Their porosity level though was ~ 57%, which is near the intersection point found here (porosity ≈ 55%) where elastic moduli were similar between lattice and staggered structures. Further, Pierantozzi et al. [5] found no significant differences between the elastic moduli of lattice and staggered scaffolds of PCL/20% HAp and PCL/20% strontium substituted-HAp at a porosity level around 40%. Accordingly, conflicting findings in the literature appear to be due to different evaluated porosities. Variations in mechanical properties of 3D printed scaffolds with changes in porosity have been attributed to column-like behavior of strand junctions when undergoing compression deformation [29]. Overall, our investigated scaffolds exhibited compressive elastic moduli in range of values consistent with previous research, in which 3D printed PCL/nHAp scaffolds were studied for BTE [5, 36, 46]. Additionally, the typical mechanical behavior of our lattice and staggered scaffolds (Fig. 1) was in agreement with previous observations, in which 3D printed scaffolds were mechanically examined [20, 30, 47–50]. Similar mechanical response to our findings (Fig. 1) has also been reported for trabecular bone [51].

Our results indicated that mechanical properties were highly dependent on porosity, with different relationships observed for lattice and staggered structures (Fig. 2A, B). The staggered structure, in particular, showed lower mechanical properties at high levels of porosity. Rationale appears to be due to the long lengths between strands at high levels of porosity, whereby longer lengths resulted in more overall deformation due to bending [22]. Further, with high degrees of bending, the junction points are subjected to higher levels of stress, resulting in scaffold failure at load-levels lower than that required for lattice structures [22]. Conversely, with the lattice structure, strands in subsequent layers intersect at similar positions, making a solid column of material from top to the bottom of scaffold; thereby avoiding bending effects [32, 36]. Surprisingly, at low porosities the staggered structures offered comparable stiffness relative to lattice structures. This is likely attributed to bending effects being minimized with shorter strand lengths, combined with load sharing of adjacent strands, which would stiffen the overall structure. To date, there are a few finite element (FE) modelling studies pertaining to compressive load distribution throughout scaffolds with various porosity levels (58–79%) [52] as well as scaffolds with and without interlayer offset (i.e., staggered and lattice scaffolds) [32]. Thus, further research via FE modelling, digital image or volume correlation should be considered to investigate the effect of internal structure (lattice and staggered scaffolds) as a function of porosity on compressive mechanical properties for the purpose of BTE.

PCL/nHAp scaffolds studied here serve as potential substitutes for trabecular bone. Morphologically, the total porosity values of our investigated scaffolds were found to be in the range of porosity in human trabecular bone (~ 30–90% [38, 53]). Additionally, the size of pores in trabecular bone has been reported to be in scale of 1.00 mm [54], which was comparable to the higher end of pore size range obtained in our investigated scaffolds. Mechanical-wise, although a wide range of compressive elastic moduli have been reported in the literature for trabecular bone [55], lattice and staggered scaffolds examined in this study met the compressive *E* of pelvic trabecular bone (*E* ~ 40 MPa [56]) as well as vertebral trabecular bone (*E* ~ 14–165 MPa [57]). For the specific purposes of guiding BTE of trabecular bone substitutes, with the aim of acquiring a minimum compressive *E* of ~ 100 MPa (minimum targeted *E* for trabecular bone [40]), lattice and staggered scaffolds should employ porosities of ~ 50%. With regard to yield strength of trabecular bone, although (again) a wide range of values have been reported in the literature [58], examined lattice and staggered structures reached values matching vertebral trabecular bone (0.9–10 MPa [57]), as well as the minimum targeted *Sy* for trabecular bone (2 MPa [41, 57]). When using a design porosity of 50%, both lattice and staggered structures meet this minimum targeted value (lattice: *Sy* = 12 MPa; staggered: *Sy* = 8 MPa).

As noted earlier, a scaffold structure must meet mechanical property requirements as well as encourage cellular activities. In general, pore sizes larger than 0.300 mm are recommended for repairing large bone defects due to enhanced new bone and capillaries formation (i.e., vascularization) [59], and a pore size of 0.300–0.400 mm was found to be the optimal pore size for bone formation in porous blocks of hydroxyapatite [60]. In addition, in vitro and in vivo studies on PCL scaffolds have shown that the promising pore size range for bone formation was between 0.290 mm and 0.310 mm [61]. The pore size of 0.350 mm was found to be favorable in poly (propylene fumarate)/diethyl fumarate scaffolds in terms of cell proliferation [62]. Our investigated lattice and staggered scaffolds with *E* ≥ 100 MPa (i.e., porosities less than or equal to 50%) showed the pore sizes ranging from ~ 0.280 mm to ~ 0.390 mm, which meet these criteria. However, further research is needed identifying the specific pore size and porosity, which offer optimum mechanical properties and cellular activities.

This study has specific strengths and limitations requiring considerations. First, this study evaluated multiple porosities of lattice and staggered structures, which helped explain conflicting findings in the literature and provided valuable design information for researchers creating tissue-engineered constructs. Second, this study verified scaffold design parameters and composition using SEM and SEM/EDX. With regard to limitations, cellular activities on scaffolds such as cell viability and capability of cells to secrete mineralized matrix are as important as structural integrity but were not assessed in this study; this is an aim of future research. Next, the key aim of this study was to assess apparent modulus and yield strength under quasi-static compressive loading. Future work is necessary to assess other mechanical properties (e.g., elastic recovery, dynamic behavior).

## Conclusions

This study found that porosity explained a high degree of variation in elastic moduli and yield strength for PCL/nHAp scaffolds with staggered and lattice structures. Our results also indicated different relationships between mechanical properties and porosities with the staggered and lattice structures. For elastic moduli, the two relationships intersected (porosity = 55%) such that the lattice structures exhibited higher moduli with porosity values greater than the intersection point; vice versa for the staggered structures. For yield strength, the lattice structure exhibited higher strength at all porosities. Taken together, this study demonstrates that scaffolds printed from PCL/30% (wt.) nHAp with lattice and staggered structure offer promise for treating trabecular bone defects.

## Methods

### Raw materials

PCL pellets (Mw = 40,000–50,000, Mn = 45,000) and nHAp powder (particle size < 200 nm) [63] were purchased from Sigma-Aldrich Canada Co.

### Preparation of composite material

A solvent-free melt blending technique was used to prepare the composite material, consisting of PCL and 30% (wt.) nHAp. As per our previous study [63], PCL pellets were first melted in a beaker at a temperature of 120 °C. nHAp powder was then slowly added and stirred to make a homogenous mixture. The resulting slurry was left to solidify and cut into small pieces for 3D printing.

### Design and printing of composite scaffolds

Square scaffolds (10 mm × 10 mm) were designed using computer-assisted design (CAD) software. To create the computer model of a square scaffold for printing, the scaffold was designed using Magics 13 EnvisionTEC software. Afterwards, slicing the designed scaffold was performed using Bioplotter RP software. A needle with an internal diameter (*d*) of 0.510 mm was applied and the layer thickness was set at 80% of the strand diameter (i.e., 0.408 mm) for gravitational spreading considerations [63]. A distance between two adjacent strands (*L*) of 1.00 mm was used; this distance was measured from the center of strands. PCL/30% (wt.) nHAp scaffolds were then printed using an extrusion-based 3D Bioplotter Manufacturer Series system (EnvisionTEC GmbH) equipped with a high-temperature printing head and a nozzle extruding the material onto a printing bed. Optimized printing parameters were as follows: nozzle temperature = 120 °C; printing bed temperature = 37 °C; nozzle offset (distance between nozzle rim and printing bed) of 0.1 mm; printing pressure = 5 bar; print speed = 1 mm/s. These printing parameters offered strand diameters and pore sizes most closely matching (~ 2% difference) the CAD model (Additional file 2: Tables S1–S2).

**Fig. 4:**
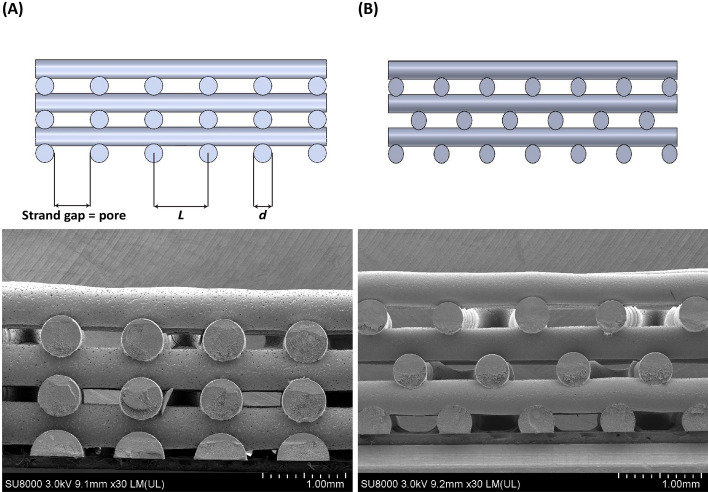
Representative SEM images of scaffolds with their schematic illustrations: **A** lattice side, **B** staggered side. The gap between the strands refers to the pore size associated with each scaffold. *L* refers to the distance between strands and *d* refers to strand diameter

Following the CAD model, PCL/30% (wt.) nHAp scaffolds with no interlayer offset value, hereinafter called lattice structure (Fig. 4A), were fabricated with a 0°/90° lay-down pattern onto a printing bed with a temperature of 37 °C. To fabricate scaffolds with an interlayer offset, referred to as staggered structure, the first and second layers were printed with 0°/90° lay-down pattern, then third and fourth layers were printed with an interlayer offset value equal to half the distance between strands (50% interlayer offset value) in both 0° and 90° directions. The fifth and sixth layers were also printed following the similar pattern (Fig. 4B). Due to 3D printer malfunction, a portion of the staggered scaffolds were 3D printed with 5 layers instead of the intended 6 layers.

### Morphological analysis

#### Pore size

Although printability was high, pore size of different scaffolds was verified using scanning electron microscopy (SEM) (Hitachi SU8010), which is a standard technique in the field. Each scaffold was coated with 10 nm of gold (Quorum Q150TES Sputter Coater) prior to the SEM analysis and then scanned under high vacuum at an accelerating voltage of 3.0 kV. The SEM images from various sites of cross sections were taken (Fig. 4A, B). All scaffolds showed porous structures with well-defined geometry, quadrangular and interconnected pores, as well as good bond between layers. ImageJ [64] was used with SEM images to measure pore size, determined via the largest diameter circle, which fit between the strands. For this study, the range of pore sizes were 0.280–0.991 mm for lattice structures and 0.280–1.086 mm for staggered structures.

#### Porosity

Porosity of both lattice and staggered scaffolds were first designed via CAD and a mathematical model in order to achieve a porosity range between 40 and 70% (Additional file 3: Table S3). Following printing, basic laboratory tests were used to characterize apparent porosity of actual 3D printed scaffolds via Eq. (1) [14, 34]:

1 $$Apparent\,porosity\,\left( \% \right)\, = \,\left( {1\, - \,\frac{{\rho_{app.} }}{{\rho_{s} }}} \right)\, \times \,100,$$

where *ρ*_*app.*_ and *ρ*_*s*_ are the apparent density of scaffold and the strand density, respectively. Apparent density was calculated as the scaffold mass divided by the volume of scaffold (M/V) and the strand density, which is composed of PCL and nHAp, was calculated through the rule of mixture using Eq. (2):

2 $$\rho_{s} \, = \,X_{PCL} \rho_{PCL} \, + \,X_{nHAp} \rho_{nHAp} ,$$

where the *X*_*PCL*_ and *X*_*nHAp*_ are the weight fraction of PCL (70%) and nHAp (30%), respectively, while *ρ*_*PCL*_ and *ρ*_*nHAp*_ refer to the density of PCL (1.145 g/cm^3^) and nHAp (3.14 g/cm^3^), respectively [5, 34]. The measured apparent porosity of scaffolds (total samples = 95) was comparable to the designed porosity. The average difference between designed and measured porosities for lattice and staggered structure was 7.6% and 0.65%, respectively (Additional file 3: Table S3).

In this study a pore size of 0 mm (i.e., length between strands = strand diameter) corresponds with a porosity of ~ 22%, which represents the lowest possible porosity (porosity is not equal to 0% due to the use of circular strands in the design). Of note, we did not create scaffolds with very low (i.e., lower than 35%) and very high porosity values (i.e., higher than 70%). For very low porosities, diffusion of adjacent layers occurred; whereas, with high porosities structural integrity diminished. Therefore, we restricted our study to porosity levels between 40 and 70%.

### Compositional verification

**Fig. 5:**
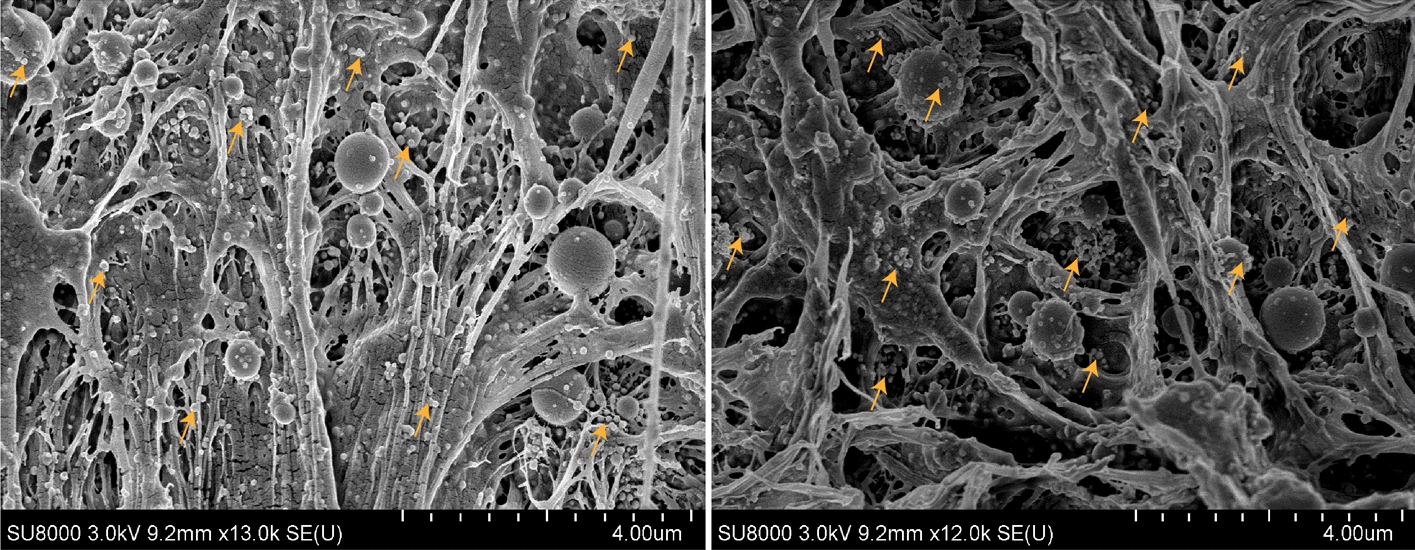
Representative SEM images displaying distribution of nHAp particles within the polymeric matrix of PCL. The small spherical structures are nHAp particles (a number of particles are marked with arrows for reference)

The presence of nHAp particles within the PCL was examined and verified using the SEM images taken from cross-sectional views. Representative SEM images displayed in Fig. 5 demonstrate that nHAp particles were successfully embedded and well distributed within the polymeric matrix of PCL. Elemental analysis of the scaffolds was also performed using energy-dispersive X-ray spectroscopy (EDX) (Ultime Max) in conjunction with SEM. The EDX analysis confirmed the presence of nHAp particles within the PCL matrix of 3D printed scaffolds. The Ca/P atomic ratios of nHAp derived from the spectra (Additional file 4: Figure S2) were 1.82 and 1.84, respectively (Additional file 4: Table S4), which were comparable to the theoretical value of HAp (Ca/P = 1.67) [23, 65].

### Mechanical testing

Square scaffolds (10 mm × 10 mm × 2.0 mm) were mechanically tested in compression (MTS Bionix^®^ Servohydraulic Test System) with a 5-kN load cell and a crosshead speed of 1.0 mm/min [42, 63]. In regard to lattice structure, 46 scaffolds with the porosity range ~ 36–73% were tested. For staggered structure, 49 scaffolds with the porosity range ~ 38–74% were tested. The scaffolds were compressed to 75% of the total initial height. ASTM standard designation D695-15 [66] was followed with regard to testing procedures and calculations of mechanical properties. Apparent elastic modulus (*E*) was derived from the slope of linear region of stress–strain curve in the elastic region using linear regression; yield strength (*Sy*) was defined as the point where the linear region of the stress–strain curve diverged from the stress–strain curve (Fig. 1).

### Statistical analysis

Scaffold mechanical properties were assessed as a function of porosity using linear regression for both the lattice and staggered structures (Fig. 2A, B). Overall tests for coincidence were used to determine if the modulus–porosity and strength–porosity relationships for the lattice and staggered structures were similar [67]. This approach uses F-test statistics to assess whether fitting separate regression curves to lattice and staggered datasets more effectively predicts mechanical properties than a single regression curve fit to both datasets. GraphPad Prism 9.3.0 was used to complete the statistical analyses. The significance level was set at *p* ≤ 0.05.

## Supplementary Information

**Additional file 1: Figure S1. **Mechanical properties of 5-layer scaffolds vs. 6-layer scaffolds as a function of porosity, A) *E* vs. porosity (*p*-value for coincidence test = 0.97), B) *Sy* vs. porosity (*p*-value for coincidence test = 0.09).

**Additional file 2: Table S1.** Influence of printing pressure on printability of composite scaffolds. Bolded indicates significant difference (*p* ≤ 0.05) of the printed structure when compared to the CAD structure. **Table S2.** Influence of nozzle speed on printability of composite scaffolds. Bolded indicates significant difference (*p* ≤ 0.05) of the printed structure when compared to the CAD structure.

**Additional file 3: Table S3.** Apparent porosities of lattice and staggered scaffolds along with the %difference between designed and measured porosities for each structure.

**Additional file 4: Figure S2.** EDX spectra in conjunction with SEM showing the elements present in the composite scaffolds of PCL/30% (wt.) nHAp. **Table S4.** Elemental analysis (atomic%) from composite scaffolds of PCL/30% (wt.) nHAp.

## Data Availability

The dataset(s) supporting the conclusions of this article is(are) included within the article [and its additional file(s)].

## Abbreviations

3D: Three-dimensional
PCL: Poly-ε-caprolactone
HAp: Hydroxyapatite
nHAp: Nano-hydroxyapatite
BTE: Bone tissue engineering
CAD: Computer-assisted design
SEM: Scanning electron microscopy
EDX: Energy-dispersive X-ray spectroscopy
PLA: Polylactic acid
PEG: Polyethylene glycol
