# Supplementary material for: Printing tissue-engineered scaffolds made of polycaprolactone and nano-hydroxyapatite with mechanical properties appropriate for trabecular bone substitutes
Source: Biomed Eng Online. 2023 Jul 20;22:73. doi: 10.1186/s12938-023-01135-6 (PMC10360269; doi:10.1186/s12938-023-01135-6)
Supplement: Supplementary file 1 — Additional file 1: Figure S1. Mechanical properties of 5-layer scaffolds vs. 6-layer scaffolds as a function of porosity, A) E vs. porosity (p-value for coincidence test = 0.97), B) Sy vs. porosity (p-value for coincidence test = 0.09). [file 12938_2023_1135_MOESM1_ESM.docx]

(A)

(B)

Figure S1. Mechanical properties of 5-layer scaffolds vs. 6-layer scaffolds as a function of porosity, A) *E* vs. porosity (*p*-value for coincidence test = 0.97), B) *Sy* vs. porosity (*p*-value for coincidence test = 0.09)
