# Supplementary material for: Printing tissue-engineered scaffolds made of polycaprolactone and nano-hydroxyapatite with mechanical properties appropriate for trabecular bone substitutes
Source: Biomed Eng Online. 2023 Jul 20;22:73. doi: 10.1186/s12938-023-01135-6 (PMC10360269; doi:10.1186/s12938-023-01135-6)
Supplement: Supplementary file 2 — Additional file 2: Table S1. Influence of printing pressure on printability of composite scaffolds. Bolded indicates significant difference (p ≤ 0.05) of the printed structure when compared to the CAD structure. Table S2. Influence of nozzle speed on printability of composite scaffolds. Bolded indicates significant difference (p ≤ 0.05) of the printed structure when compared to the CAD structure. [file 12938_2023_1135_MOESM2_ESM.docx]

**Printability:**

To assess the accuracy of the printed scaffolds relative to the CAD models (*i.e*., printability), square scaffolds with two printing layers were fabricated. CAD modelled strand diameter and pore size were 0.510 mm and 0.490 mm, respectively. Optimized printing parameters, based upon pilot studies, were as follows: nozzle temperature = 120 ˚C; printing bed temperature = 37 ˚C; nozzle offset (distance between nozzle rim and printing bed) of 0.1 mm.

Different printing pressures and nozzle speeds were applied to assess their effect on printability. Scaffolds were printed with pressures between 4 to 7 bar at a constant nozzle speed of 1 mm/s. Next, scaffolds were printed with nozzle speeds between 0.4 to 2.1 mm/s at a constant printing pressure of 5 bar. Three samples were printed for each experimental group. Scaffolds were imaged with a camera under an optical microscope (Leica DMIL) at 100x magnification. Printed strand diameter and pore size were measured using ImageJ software [1] and compared to CAD modelled parameters. Strand printability was also characterized via Eq. (S1):

$Strand printability=1-\frac{d_{CAD}-d_{p}}{d_{CAD}}$ (S1)

where *d_CAD_* is the CAD value for strand diameter and *d_P_* is the printed strand diameter. Here a value of 1 indicates optimum printability [2].

Scaffolds printed at pressures of 4, 6 and 7 bar (constant nozzle speed of 1 mm/s) exhibited different strand diameters and pore sizes versus the designed values (Table S1). Similarly, scaffolds printed with nozzle speeds of 0.4-0.8 mm/s and 1.1-2.1 mm/s (constant pressure of 5 bar) exhibited different strand diameters and pore sizes versus the designed values (Table S2). No differences were noted with a printing pressure of 5 bar and nozzle speed of 1 mm/s. Also, strand printability did not differ from 1 with these printing parameters.

Table S1. Influence of printing pressure on printability of composite scaffolds. Bolded indicates significant difference (*p* ≤ 0.05) of the printed structure when compared to the CAD structure.

| Pressure (bar) | Speed (mm/s) | *d* (mm) | Pore size (mm) | Strand printability |
| --- | --- | --- | --- | --- |
| **4** | **1** | **0.394±0.025** | **0.538±0.009** | **0.77±0.048** |
| 5 | 1 | 0.522±0.023 | 0.477±0.016 | 1.02±0.044 |
| **6** | **1** | **0.565±0.022** | **0.433±0.009** | **1.11±0.044** |
| **7** | **1** | **0.633±0.027** | **0.342±0.01** | **1.24±0.053** |

Table S2. Influence of nozzle speed on printability of composite scaffolds. Bolded indicates significant difference (*p* ≤ 0.05) of the printed structure when compared to the CAD structure.

| Pressure (bar) | Speed (mm/s) | *d* (mm) | Pore size (mm) | Strand printability |
| --- | --- | --- | --- | --- |
| 5 | 0.4 | NA | NA | NA |
| **5** | **0.6** | **0.731±0.05** | **0.209±0.04** | **1.43±0.09** |
| **5** | **0.8** | **0.610±0.03** | **0.340±0.03** | **1.20±0.07** |
| 5 | 1 | 0.522±0.023 | 0.477±0.016 | 1.02±0.044 |
| **5** | **1.1** | **0.441±0.025** | **0.526±0.030** | **0.87±0.05** |
| **5** | **1.3** | **0.401±0.005** | **0.558±0.034** | **0.79±0.01** |
| **5** | **1.5** | **0.376±0.007** | **0.582±0.014** | **0.74±0.01** |
| **5** | **1.7** | **0.400±0.02** | **0.570±0.01** | **0.78±0.03** |
| **5** | **1.9** | **0.358±0.01** | **0.605±0.01** | **0.70±0.02** |
| **5** | **2.1** | **0.357±0.01** | **0.651±0.02** | **0.70±0.03** |

**References**

[1] Rasband WS. ImageJ, U.S. National Institutes of Health, Bethesda, Maryland, USA, imagej.nih.gov/ij/,1997–2012.

[2] S. Naghieh, M.D. Sarker, N.K. Sharma, Z. Barhoumi, X. Chen, Printability of 3D Printed Hydrogel Scaffolds: Influence of Hydrogel Composition and Printing Parameters, Appl. Sci. . 10 (2020). https://doi.org/10.3390/app10010292.
