# Supplementary material for: Printing tissue-engineered scaffolds made of polycaprolactone and nano-hydroxyapatite with mechanical properties appropriate for trabecular bone substitutes
Source: Biomed Eng Online. 2023 Jul 20;22:73. doi: 10.1186/s12938-023-01135-6 (PMC10360269; doi:10.1186/s12938-023-01135-6)
Supplement: Supplementary file 3 — Additional file 3: Table S3. Apparent porosities of lattice and staggered scaffolds along with the %difference between designed and measured porosities for each structure. [file 12938_2023_1135_MOESM3_ESM.docx]

**Designed porosity:**


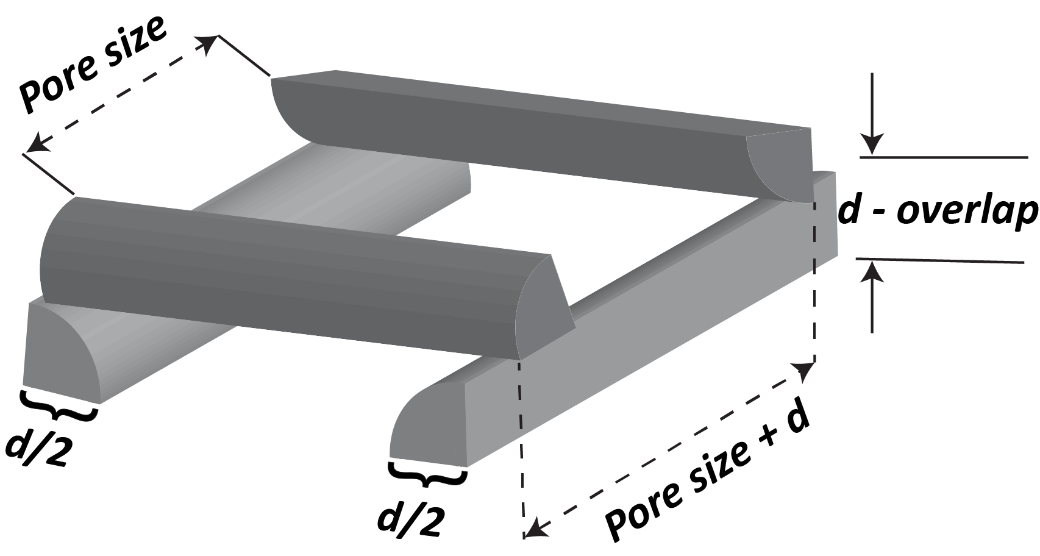


Porosity of scaffolds was designed via the mathematical model as follows.

$$Designed porosity=1-volume fraction=1- \frac{volume of material}{total volume}$$

$$Designed porosity=1- \frac{\left( \frac{\pi\times d^{2}}{4} \right)\times(pore size+d)}{{(pore size+d)}^{2}\times(d-\Delta_{0})}$$

The term Δ_0_ pertains to the overlap in relation to the strand diameter. Of note, during printing, each 3D printed strand fused to the strand in the underlying layer. This was due to the impact of gravity, which spreads the strands, as well as the temperature gradient between the extruded material from the nozzle tip and the pre-printed material on printing bed [1]. As a result, we considered this parameter in our mathematical model in order to improve predictions between the designed porosity and measured porosity of fabricated scaffolds. In this study, overlap was considered 10% of strand diameter. Also, different spacing between the strands (pore size + *d*) and strand diameters were used to design scaffolds with porosities ranging from 40% to 70%.

Table S3. Apparent porosities of lattice and staggered scaffolds along with the %difference between designed and measured porosities for each structure

| **Lattice-designed porosity (%)** | **Lattice-measured porosity (%)** | **%difference** | **Staggered-designed porosity (%)** | **Staggered-measured porosity (%)** | **%difference** |
| --- | --- | --- | --- | --- | --- |
| 39.79 | 36.30 | -8.76 | 41.79 | 38.37 | -8.19 |
| 39.79 | 37.47 | -5.82 | 47.64 | 38.80 | -18.56 |
| 39.79 | 41.03 | 3.13 | 41.79 | 39.25 | -6.09 |
| 39.79 | 41.59 | 4.53 | 47.64 | 39.25 | -17.61 |
| 39.79 | 41.80 | 5.06 | 41.79 | 39.42 | -5.68 |
| 47.64 | 41.91 | -12.03 | 47.64 | 39.76 | -16.54 |
| 47.64 | 43.08 | -9.57 | 47.64 | 40.35 | -15.30 |
| 47.64 | 43.76 | -8.14 | 41.79 | 40.70 | -2.62 |
| 47.64 | 44.90 | -5.75 | 41.79 | 41.52 | -0.65 |
| 47.64 | 45.70 | -4.07 | 41.79 | 41.91 | 0.28 |
| 47.64 | 48.03 | 0.82 | 41.79 | 42.07 | 0.66 |
| 47.64 | 48.45 | 1.70 | 41.79 | 42.17 | 0.90 |
| 47.64 | 49.38 | 3.65 | 47.64 | 43.10 | -9.53 |
| 47.64 | 50.24 | 5.46 | 50.44 | 45.48 | -9.83 |
| 47.64 | 53.34 | 11.96 | 50.44 | 47.77 | -5.29 |
| 47.64 | 53.81 | 12.95 | 50.44 | 48.82 | -3.21 |
| 60.73 | 57.46 | -5.38 | 49.56 | 48.82 | -1.49 |
| 60.73 | 58.29 | -4.02 | 50.44 | 48.99 | -2.87 |
| 60.73 | 58.31 | -3.98 | 49.56 | 49.48 | -0.16 |
| 60.73 | 58.37 | -3.89 | 49.56 | 49.57 | 0.02 |
| 60.73 | 59.93 | -1.32 | 49.56 | 49.71 | 0.30 |
| 60.73 | 60.19 | -0.89 | 49.56 | 50.91 | 2.72 |
| 52.00 | 60.37 | 16.09 | 49.56 | 50.95 | 2.80 |
| 52.00 | 61.47 | 18.20 | 50.44 | 50.98 | 1.07 |
| 52.00 | 61.63 | 18.51 | 51.13 | 51.49 | 0.70 |
| 52.00 | 62.17 | 19.55 | 51.13 | 51.70 | 1.11 |
| 52.00 | 62.42 | 20.03 | 51.13 | 51.80 | 1.31 |
| 52.00 | 62.49 | 20.17 | 51.13 | 52.96 | 3.58 |
| 52.00 | 62.52 | 20.22 | 51.13 | 53.67 | 4.97 |
| 52.00 | 62.61 | 20.40 | 51.13 | 54.12 | 5.85 |
| 52.00 | 62.84 | 20.84 | 52.00 | 55.07 | 5.90 |
| 52.00 | 62.88 | 20.92 | 52.00 | 56.63 | 8.90 |
| 65.09 | 64.11 | -1.51 | 52.00 | 57.16 | 9.92 |
| 60.73 | 64.84 | 6.77 | 52.00 | 57.46 | 10.49 |
| 60.73 | 64.89 | 6.85 | 60.73 | 57.74 | -4.92 |
| 60.73 | 64.95 | 6.95 | 60.73 | 57.84 | -4.76 |
| 65.09 | 65.02 | -0.11 | 60.73 | 58.39 | -3.85 |
| 60.73 | 65.26 | 7.46 | 52.00 | 59.02 | 13.49 |
| 65.09 | 65.59 | 0.76 | 60.73 | 59.14 | -2.62 |
| 65.09 | 65.80 | 1.09 | 60.73 | 59.33 | -2.31 |
| 65.09 | 66.99 | 2.91 | 52.00 | 60.62 | 16.57 |
| 68.00 | 72.48 | 6.58 | 60.73 | 61.74 | 1.66 |
| 68.00 | 72.62 | 6.79 | 60.73 | 63.65 | 4.81 |
| 68.00 | 72.64 | 6.82 | 60.73 | 63.87 | 5.17 |
| 68.00 | 72.95 | 7.28 | 65.09 | 66.33 | 1.90 |
| 68.00 | 73.29 | 7.78 | 60.73 | 66.63 | 9.71 |
|  |  |  | 65.09 | 69.94 | 7.45 |
|  |  |  | 65.09 | 71.88 | 10.43 |
|  |  |  | 65.09 | 73.55 | 12.99 |

**References**

[1] D.X.B. Chen, Extrusion Bioprinting of Scaffolds for Tissue Engineering Applications, Springer International Publishing AG, Switzerland, 2019.
