# Supplementary material for: Printing tissue-engineered scaffolds made of polycaprolactone and nano-hydroxyapatite with mechanical properties appropriate for trabecular bone substitutes
Source: Biomed Eng Online. 2023 Jul 20;22:73. doi: 10.1186/s12938-023-01135-6 (PMC10360269; doi:10.1186/s12938-023-01135-6)
Supplement: Supplementary file 4 — Additional file 4: Figure S2. EDX spectra in conjunction with SEM showing the elements present in the composite scaffolds of PCL/30% (wt.) nHAp. Table S4. Elemental analysis (atomic%) from composite scaffolds of PCL/30% (wt.) nHAp. [file 12938_2023_1135_MOESM4_ESM.docx]

**Compositional Verification:**

Elemental analysis of the scaffolds was performed using energy-dispersive x-ray spectroscopy (EDX) (Ultime Max) in conjunction with SEM (Figure S2). The EDX analysis confirmed the presence of nHAp particles within the PCL matrix of 3D printed scaffolds. The Ca/P atomic ratios of nHAp derived from spectrum 10 and spectrum 11 (Table S4) were 1.82 and 1.84, respectively, which were comparable to the theoretical value of HAp (Ca/P=1.67).


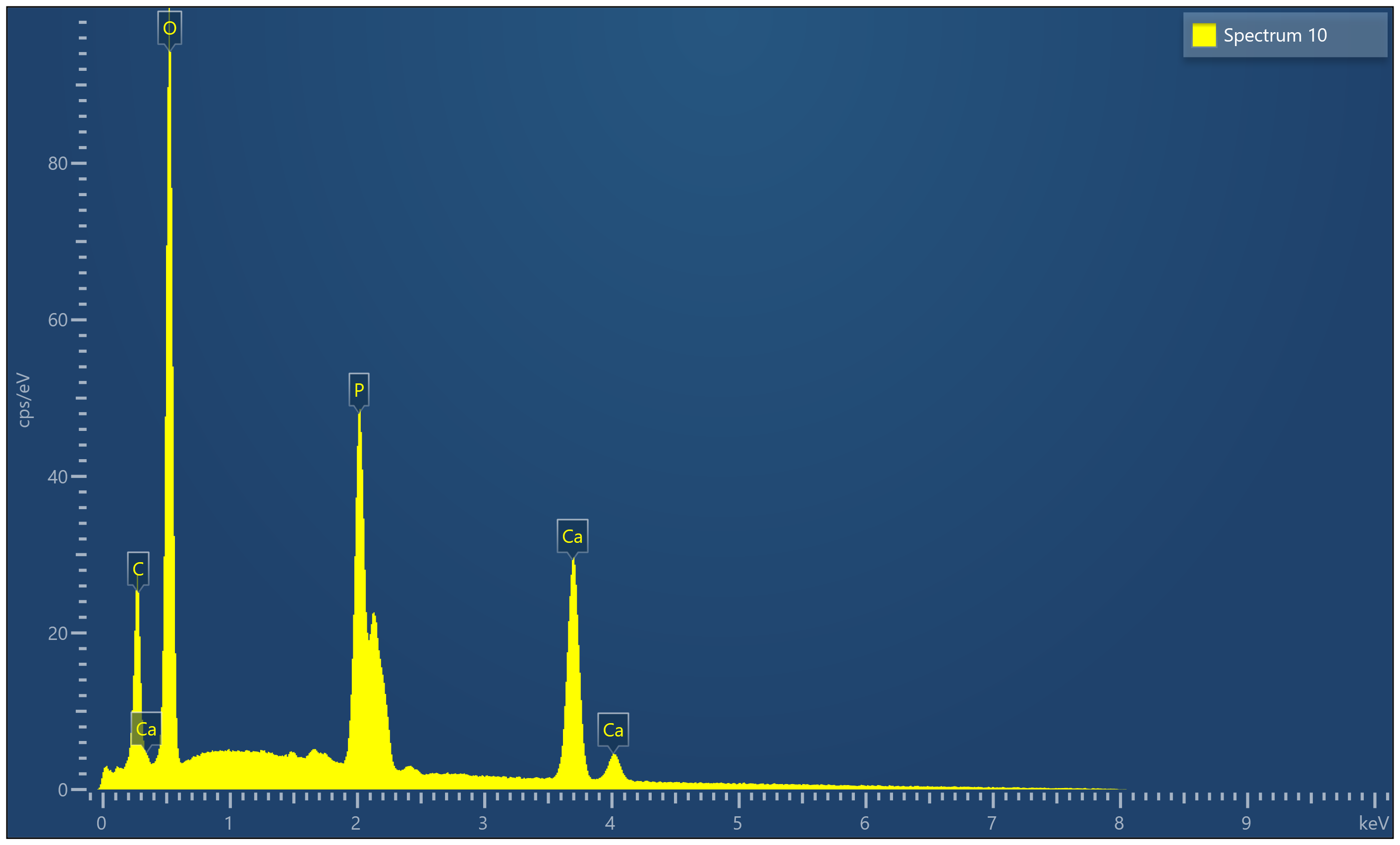

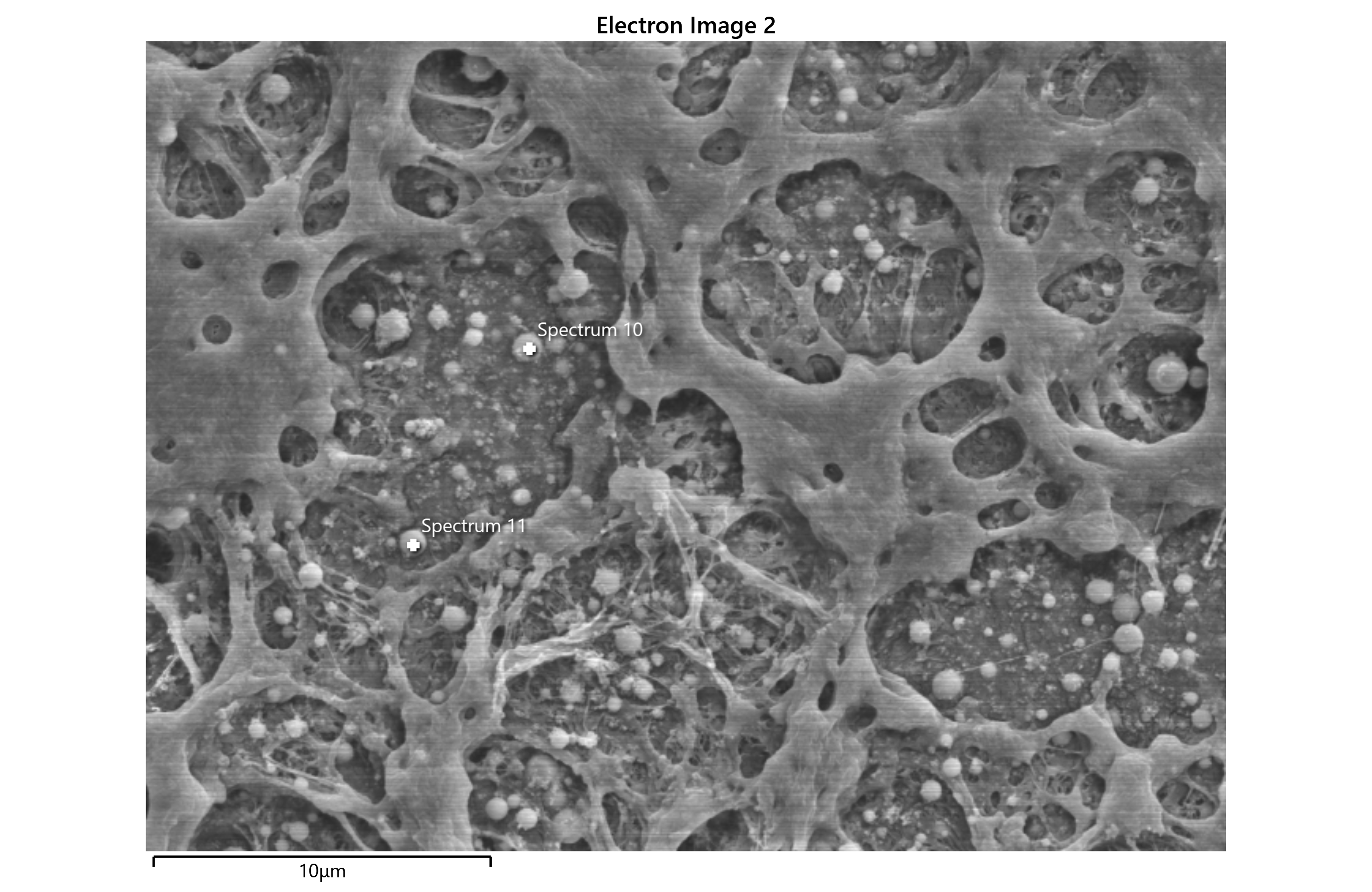

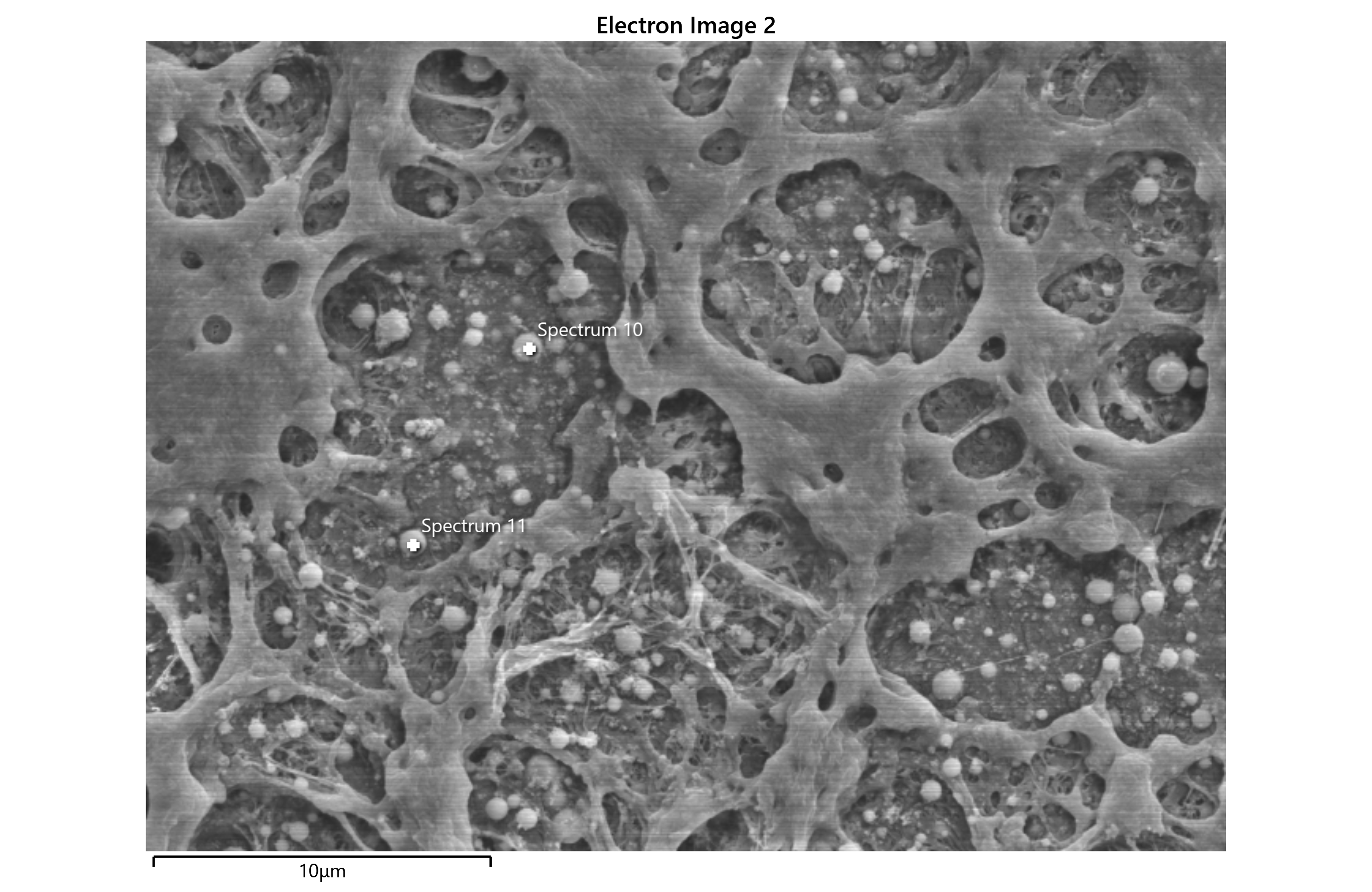

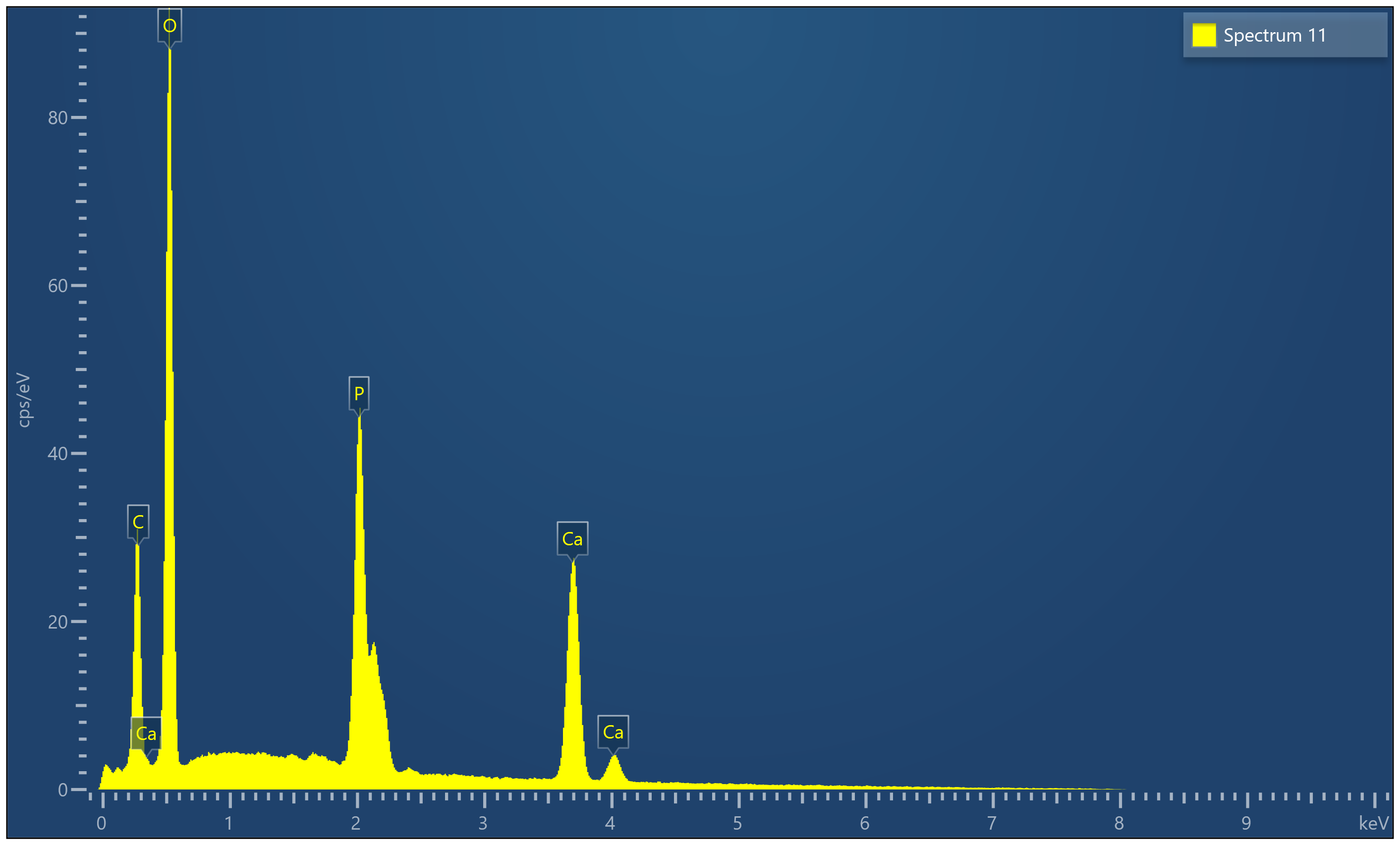

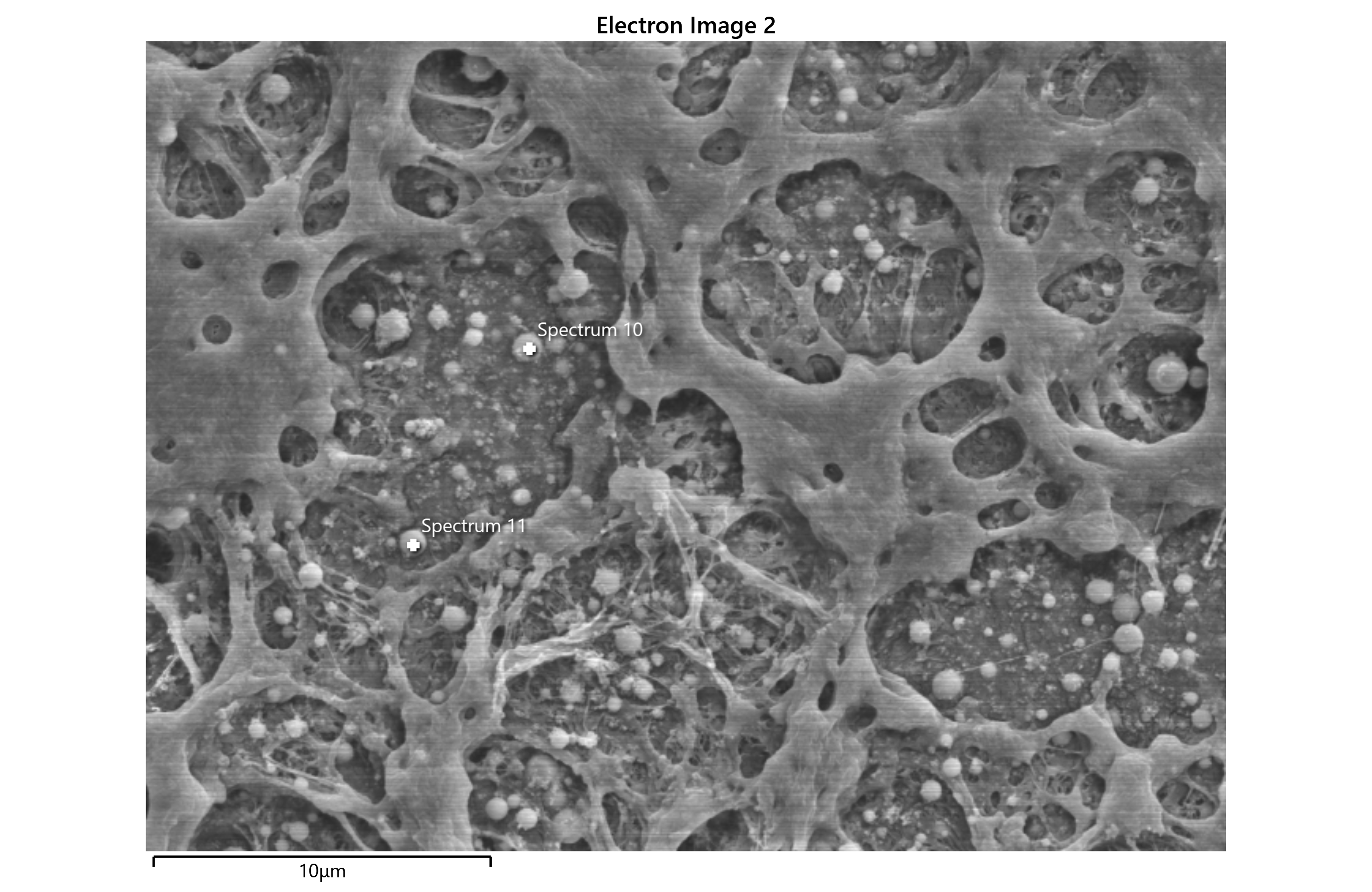

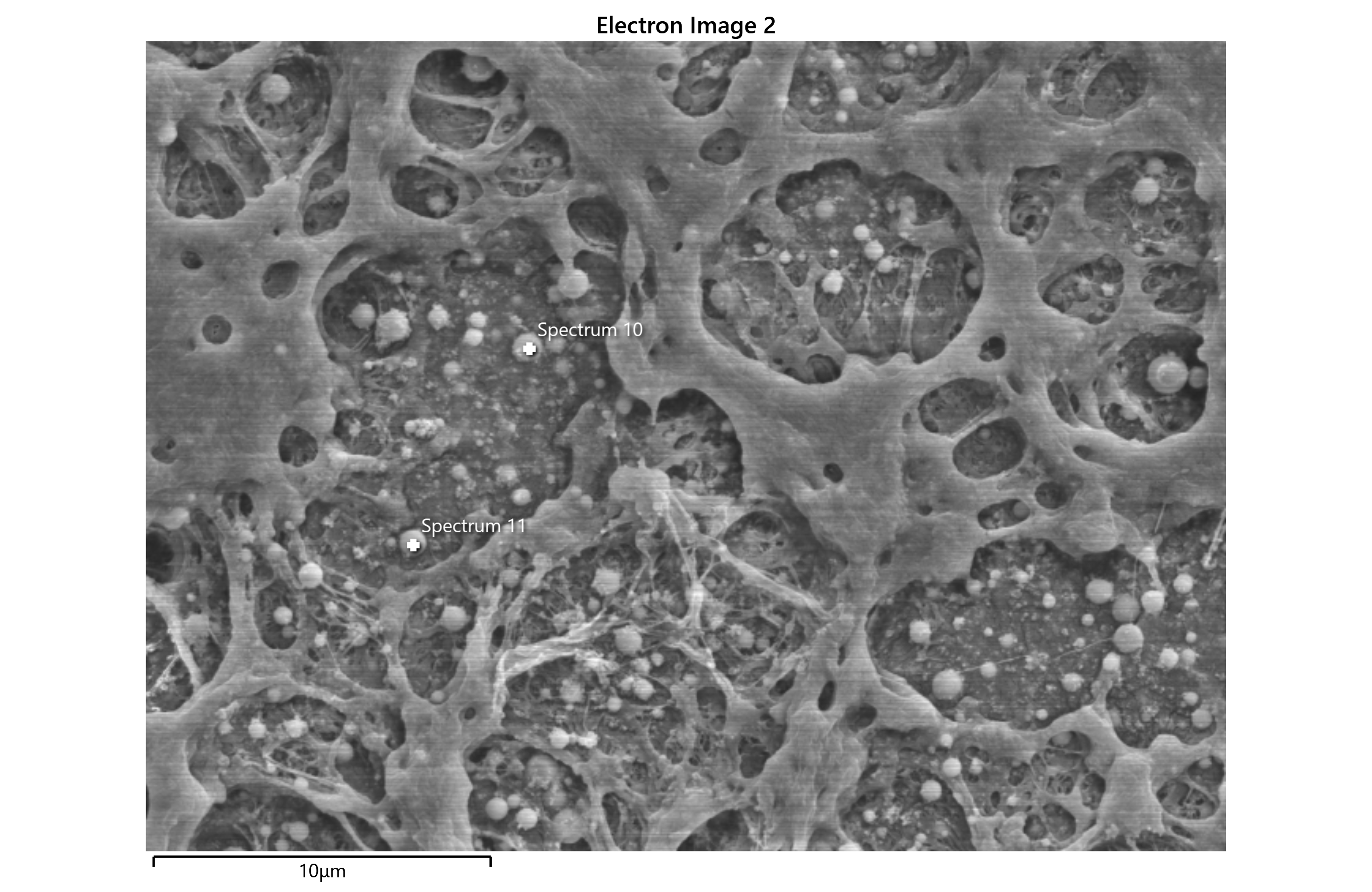


Figure S2. EDX spectra in conjunction with SEM showing the elements present in the composite scaffolds of PCL/30% (wt.) nHAp

Table S4. Elemental analysis (atomic%) from composite scaffolds of PCL/30% (wt.) nHAp

| **Elements** | **Spectrum 10 (atomic%)** | **Spectrum 11 (atomic%)** |
| --- | --- | --- |
| C | 19.75 | 23.84 |
| O | 55.30 | 52.48 |
| P | 8.84 | 8.33 |
| Ca | 16.11 | 15.36 |
| Total | 100.00 | 100.00 |
